# Sulfate Freeze–Thaw Resistance of Magnesium Potassium Phosphate Cement Mortar according to Hydration Age

**DOI:** 10.3390/ma15124192

**Published:** 2022-06-13

**Authors:** Rong-Jian Ji, Tao Li, Jian-Ming Yang, Jun Xu

**Affiliations:** 1 Yangzhou Polytechnic Institute, College of Architecture Engineering, Yangzhou 225127, China; 2 School of Civil Engineering, San Jiang University, Nanjing 210012, China; yglitaotao@163.com (T.L.); yjm_kk@163.com (J.-M.Y.); 3 College of Civil Engineering and Architecture, Jiangsu University of Science and Technology, Zhenjiang 212000, China; xujun@just.edu.cn

**Keywords:** magnesium potassium phosphate cement, hydration age, freeze–thaw resistance, sulfate corrosion, strength, volume deformation, water absorption

## Abstract

Concrete structures can be degraded by exposure to environmental stressors such as freeze–thaw cycling and salt corrosion. Magnesium potassium phosphate cement (MKPC) mortar is useful for the rapid repair of such structures but must acquire environmental resistance rapidly. In this study, the freeze–thaw resistance of MKPC mortar specimens of different hydration ages was tested in water and a 5% Na_2_SO_4_ solution. The strength, volume deformation, and water absorption rates were compared with those of full-age MKPC mortar specimens (28 d). The phase composition and microscopic morphology of the MKPC mortar specimens before and after corrosion were observed, and the corrosion-resistance mechanism was analyzed. After 225 freeze–thaw cycles in water and sulfate solution, the strength residual rates of the early-age specimen (1 d) were higher than those of the full-age specimen (28 d). The degree of strength attenuation in the 1 d specimen was lower in the sulfate environment than in the water environment. After 225 freeze–thaw cycles, the volume expansion rates of 1 d specimens in water or sulfate were 0.487% and 0.518%, respectively, while those of 28 d specimens were 0.963% and 1.308%. The comparison shows that the 1 d specimen had significantly better deformation resistance under freeze–thaw than the 28 d specimen. After 225 freeze–thaw cycles, the water absorption rates of 1 d specimens were 1.95% and 1.64% in water and sulfate solution, respectively, while those of 28 d specimens were 2.20% and 1.83%. This indicates that freeze–thaw cycling has a greater effect on the pore structure of fully aged mortar than on early-age mortar (1 d). Therefore, MKPC mortar is suitable for the rapid repair of concrete structures in harsh environments. The results form a theoretical basis for winter emergency repair projects. They also further the understanding of the application of MKPC-based materials in extreme environments.

## 1. Introduction

Concrete materials are being used in increasingly complex environments that may have unpredictable impacts on their durability. For example, the durability of concrete structures used in ports is affected by multiple environmental factors, such as humidity, temperature, and chloride corrosion. In deep underground engineering, concrete is affected by ground stress and sulfate corrosion. Salt corrosion and freeze–thaw cycling are two of the main threats to concrete structures. In northern China, combinations of these factors often cause the deterioration of concrete structures [1,2]. Compared to replacing damaged structures, timely repair is considered a more economical and environmentally friendly solution. Damage to highways, bridge decks, and port facilities must be repaired promptly to avoid impacting transportation. In northern China, the ambient temperature falls below 0 °C for an extended duration in winter. Under low-temperature conditions, conventional repair materials may take an excessively long time to set and harden, or may even fail, making it difficult to rapidly repair cement or concrete pavement [3,4].

Magnesium phosphate cement (MPC) is a new type of inorganic cementitious material that has high early strength, low shrinkage, high cohesiveness, and many other advantages that make it a desirable rapid repair material [5,6,7,8,9,10,11]. With the development of research on MPC, it is no longer limited to normal-temperature applications. Compared with other rapid-hardening and early-strength repair materials, MPC is more useful in sub-zero-temperature environments [12,13,14,15]. Jia et al. [12] studied the working performance, setting time, and mechanical properties of MPC in an ice and snow environment. The rapid reaction of lightly burned MgO releases a large amount of heat that rapidly melts ice particles to form water. MPC can be prepared at −20 °C. When the content of lightly burned magnesia to replace dead burned magnesia is 8%, MPC can attain a strength of 32 MPa after 2 h. Wang [13] explored the strength development of MPC mortar under a sub-zero-temperature environment. The results show that the compressive strength of MPC mortar cured at −20 °C for 3 h can reach 17 MPa. Lai [14] explored the strength development of MPC cured at −20 °C. Compared with MPC cured at room temperature, the matrix strength at −20 °C was reduced, and its 1 d strength was still close to 50 MPa. Yang et al. [15] studied the variation in MPC setting time with curing temperature, finding that the higher the temperature, the shorter the setting time. The setting time of MPC at −10 °C can be controlled within 1 h, making it suitable for rapid repair in low-temperature environments. In summary, the performance of MPC in low-temperature environments has received basic research. Compared with other rapid-hardening and early-strength repair materials, MPC has better performance at low temperatures. However, its durability has an important influence on the durability of the concrete structure it is used to repair and, therefore, warrants in-depth study.

There have been recent experimental studies on the water corrosion, salt corrosion, and freeze–thaw resistance of MPC systems [16,17,18,19,20,21,22]. Studies on the anti-salt freezing performance of MPC mortar have used NaCl solutions as the corrosion solution and have confirmed that MPC mortar has suitable anti-NaCl salt freezing and denudation performance [17,22]. However, in addition to chlorides, sulfates are prevalent in environments such as oceans, salt lakes, and groundwater. In saline-alkali and tidal areas, sulfate corrosion is an important cause of concrete deterioration [2,23]. Existing long-term salt immersion tests have confirmed that sulfate corrosion is more damaging to MPC-based materials than chloride salt corrosion [9,19]. When used for concrete repair, MPC-based materials mostly comprise MPC mortar and aggregate; however, the freeze–thaw resistance of MPC mortar-containing aggregate in sulfate solution has not been studied. At present, research on frost resistance has mainly focused on MPC-based materials with a hydration age ≥28 days. However, for rapid repairs, the frost resistance of MPC at an early age is equally important. According to the different phosphates used, MPC can be divided into two systems: (1) ammonium magnesium phosphate cement (MAPC) systems using ammonium dihydrogen phosphate and (2) potassium magnesium phosphate cement (MKPC) systems using potassium dihydrogen phosphate. The hydration reaction of MAPC generates a large amount of ammonia, which is harmful to health [24], while MKPC does not emit harmful gases during hydration [25], making it a better material.

In the severe-cold area of Western China, concrete structures can be degraded by exposure to environmental stressors such as freeze–thaw cycling and salt corrosion. For emergency repair projects, MKPC mortar is useful for the rapid repair of such structures but must acquire environmental resistance rapidly. Therefore, this paper studied the frost resistance of MKPC mortar at different hydration ages in water and salt solutions. The results help to understand the resistance of early-age MKPC mortar to freeze–thaw cycling in water and salt solutions. This is of great significance to its use for rapid road and structural repairs in severely cold environments.

## 2. Materials and Methods

### 2.1. Materials

MKPC mortar specimens were prepared with dead burned magnesium oxide, potassium dihydrogen phosphate, fine aggregate, composite retarder, and water. The dead burned magnesia powder was produced by the magnesia factory of Hengren Dongfanghong Hydropower Station in Liaoning Province. The industrial-grade potassium dihydrogen phosphate (KH_2_PO_4_) was provided by Lianyungang Geli Chemical Co., Ltd., (Lianyungang, China). The composite retarder was prepared in the laboratory of the project team [26]. The raw materials were all of industrial purity and were provided by the Liaoning Kuandian chemical plant. The fine aggregate used was ordinary river sand and limestone sand. Tap water was used, and the sodium sulfate used was of analytical purity.

### 2.2. Specimen Preparation

The required raw materials were calculated and weighed, and MKPC mortar was prepared in a mortar mixer. The newly mixed MKPC mortar was poured into molds of various specifications. Subsequently, the molds containing mortar were vibrated on a vibrating table, with the excess slurry scraped off. Specimens were then sealed with plastic wrap for 5 h before being demolded. The demolded specimens were placed in a curing room (temperature = 20 ± 5 °C, humidity = 60 ± 5%) until a specified age. Then, freeze–thaw tests in water and sulfate solution were conducted. To ensure that the freeze–thaw tests of MKPC mortar specimens at different hydration ages were carried out at the same time, the specific times at which the specimens were formed were carefully monitored. The experiment was designed with three kinds of hydration age MKPC mortar specimens, namely MKPC mortar specimens with a hydration age of 5 h (M-5h), MKPC mortar specimens with a hydration age of 1 d (M-1d), and MKPC mortar specimens with hydration age of 28 d (M-28d).

### 2.3. Experimental Methods

Referring to the Standard Test Method for Resistance of Concrete to Rapid Freeze and Thawing (ASTM C666-15), MKPC mortar specimens were placed in water, and 5% Na_2_SO_4_ solution, and rapid freeze–thaw tests were carried out using an HDK-5 concrete freeze–thaw testing machine. Prior to testing, MKPC specimens were saturated with their corresponding solution by placing them inside a vacuum water-saturation device for 24 h. The initial strength, water absorption, and length of the specimens were also measured before commencing the freeze–thaw test. The freeze–thaw system parameters were set as follows: the highest temperature of the center was set to 10 °C, the lowest temperature was set to −15 °C, and each cycle consisted of 3.5 h freezing and 2.5 h melting. The water and 5% Na_2_SO_4_ solutions in the sleeves were replenished with fresh solutions every 25 cycles to ensure stable concentrations of the corrosive media. After the set number of cycles was reached, the mass, deformation, flexural strength, and compressive strength of the specimens were measured.

According to the Standard Test Method for Flexural Strength of Hydraulic-Cement Mortars (ASTM C348-2008) and Standard Test Method for Compressive Strength of Hydraulic-Cement Mortars (ASTM C349-2008), the flexural and compressive strengths of the MKPC specimens (with dimensions of 40 mm × 40 mm × 160 mm) were measured using a WED-300 electronic universal testing machine. The residual strength ratio could then be obtained by comparing the flexural (compressive) strength of the MKPC specimens after n cycles of freezing and thawing in the water or salt solutions with their initial flexural (compressive) strength.

Referring to the Standard Test Method for Measurement of Rate of Absorption of Water by Hydraulic-Cement Concretes (ASTM C1585-2013), the saturated surface dry mass ($W_{n}$) of the MKPC specimens (φ Φ50 × 150 mm) subjected to n freeze–thaw cycles in water or salt solutions were measured by an electronic balance with an accuracy of 0.01 g. The specimens were then placed in a vacuum drying box at 60 °C for 48 h and then naturally cooled. The mass of the dried specimen ($W_{n}^{*}$) was weighed, and the water absorption of each MKPC specimen was calculated according to Formula (1):

(1) $$\rho_{n}=\frac{W_{n}-W_{n}^{*}}{W_{n}^{*}}\times 100\%$$

According to a modified British Standard method BS ISO 1920-8 (2009), “Testing of concrete-Determination of the drying shrinkage of concrete for samples prepared in the field or the laboratory”, the deformation of MKPC mortar specimens was tested. The deformation rate was calculated in accordance with Formula (2):

(2) $$\varepsilon_{n}=\frac{L_{n}-L_{0}}{250}\times 100\%$$

where $\varepsilon_{n}$ is the deformation rate, *L*_0_ is the length of the specimen in the saturated state after being soaked in the solution, and *L_n_* is the length of the specimen in the saturated state after *n* freeze–thaw cycles.

XRD, SEM, and TG-DTG analyses were performed on crushed MKPC samples after strength measurements were taken. First, the sample surface was cleaned to remove any attached particles and then immersed in an anhydrous ethanol solvent to prevent hydration. Before the SEM observation, the MKPC sample was taken out and cut into 3–5 mm flakes, with one side of each flake made flat and smooth. The processed sample was placed in sealed glassware and dried at room temperature. The prepared samples were placed into a gold spraying apparatus and sprayed with gold for 2 min. The microscopic morphology of the samples was observed by SEM (QUANTA 200 environmental SEM, FEI Co., Hillsboro, OR, USA). Point or area energy spectroscopy was performed on the hydration product to analyse the chemical composition of the product. Part of the sample was ground into powder and passed through a 200-mesh sieve (75 μm). The phase composition of the MKPC samples was determined by X-ray diffraction (XRD; D/max-RB X-ray diffraction instrument). In the test, the tube voltage was 40 kV, the tube current was 20 mA, the anode target material was Cu, and the scanning range was 10~80° (2θ). The scanning speed was 6°/min, the step size was 0.026°DS (divergent slit) = SS (anti-scattering slit) = 1° and RS (receiving slit) = 0.3 mm. According to the diffraction data, the phase of the sample was searched for, confirmed, and analyzed using the International Standard Diffraction Database (ICDD PDF-2, 2008). The TG-DTG tests used a TGA/DSC1 thermogravimetric analyzer (METTLER TOLEDO Co., Zurich, Switzerland). Samples were heated from 20 °C to 1000 °C at a rate of 10 °C/min. Nitrogen was used as a protective gas, and α-Al_2_O_3_ was used as the reference.

## 3. Results and Discussion

### 3.1. Material Analysis and Mix Ratio

The specific surface area of the dead burned magnesium oxide powder was 230 m^2^·kg^−1^, its average particle size was 45.26 μm, and the oxide composition based on XRF fluorescence analysis is shown in Table 1. XRF fluorescence analysis confirmed that the MgO content in the dead burned magnesium oxide powder exceeded 90%, while the SiO_2_ and CaO contents both exceeded 3%. The industrial-grade potassium dihydrogen phosphate was white columnar crystals with a main particle size of 40/380 to 60/250 (mesh/μm). The main particle size of raw materials used in the composite retarder is 60/250~80/180 (mesh/μm). The physical indexes of ordinary river sand and limestone sand are shown in Table 2.

Table 3 shows the mix proportions of the materials used to prepare the MKPC mortar. The acid-base ratio and bone-glue ratio were selected by comparison and optimization [27].

### 3.2. Flexural Strength Development

**Figure 1:**
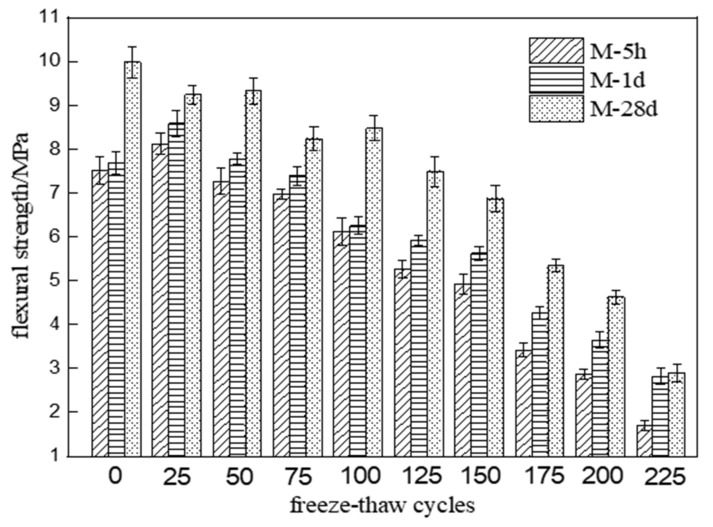
Flexural strength changes of MKPC mortar specimens with the number of freeze–thaw cycles in water (M-5h, M-1d, and M-28 refer to MKPC mortar specimens at hydration ages of 5 h, 1 d, and 28 d, respectively).

Figure 1 shows the flexural strength changes of three different MKPC mortar specimens with an increasing number of freeze–thaw cycles in water. Note that a 24 h vacuum saturation treatment was performed before the freeze–thaw test. Therefore, the actual hydration age of each specimen at the start of the freeze–thaw test is the age indicated in the figure plus 24 h. As can be seen from Figure 1, the flexural strength of M-5h and M-1d both rose to a peak value after 25 freeze–thaw cycles and then showed a fluctuating decreasing trend with further cycling. In contrast, the flexural strength of M-28d showed a continuously fluctuating decreasing trend with an increasing number of freeze–thaw cycles. The flexural strength reached 75% of its initial values after 100 cycles for M-5 and 125 cycles for M-1d. On the other hand, the flexural strengths of M-28d reached 75% of their initial values after 125 cycles. After 225 cycles, the flexural strengths of M-5h, M-1d, and M2-28d were 22.6%, 36.7%, and 29.0% of their initial values, respectively. The residual flexural strengths of M-1d and M-28d were found to be similar. Overall, the results show that the flexural strength deterioration of MKPC mortar at 1-day hydration age is close to that of MKPC mortar at 28-day hydration age under freezing and thawing conditions in water.

**Figure 2:**
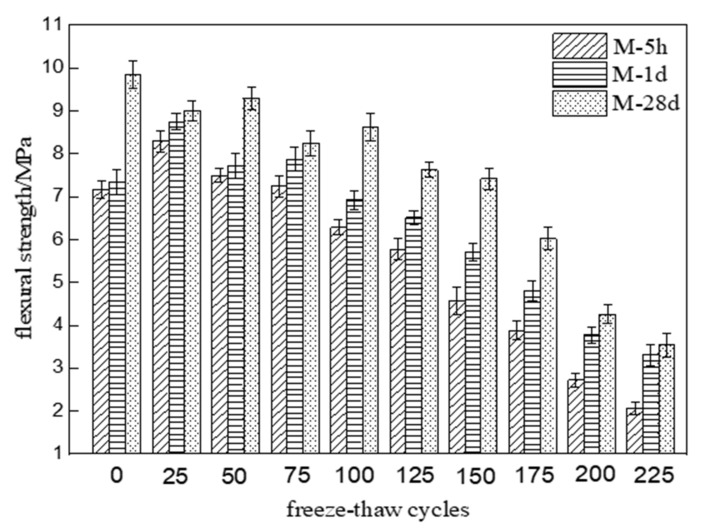
Flexural strength changes of MKPC mortar specimens with number of freeze–thaw cycles in 5% Na_2_SO_4_ solution.

Figure 2 shows the flexural strength changes of three different MKPC mortar specimens with the number of freeze–thaw cycles in a 5% Na_2_SO_4_ solution. The flexural strength of M-5h and M-1d gradually rose to a peak value after 25 freeze–thaw cycles and then showed a fluctuating decreasing trend with an increasing number of freeze–thaw cycles. In contrast, the flexural strength of M-28d showed a fluctuating decreasing trend with an increasing number of freeze–thaw cycles. The flexural strength reached 75% of its initial values after 125 cycles of M-5h and 150 cycles for M-1d. On the other hand, the flexural strengths of M-28d reached 75% of their initial values after 150 cycles. After 225 cycles, the flexural strengths of M-5h, M-1d, and M-28d were 28.9%, 45.1%, and 36.0% of their initial values, respectively. The residual flexural strengths of M-1d and M-28d were similar. According to the results of Figure 1 and Figure 2, the flexural strength deterioration degree of MKPC mortar specimens under freezing and thawing conditions in sulfate solution is lower than that in water. The flexural strength deterioration degree of MKPC mortar at 1-day hydration age is close to that of MKPC mortar at 28-day hydration age under freezing and thawing conditions.

### 3.3. Compressive Strength Development

**Figure 3:**
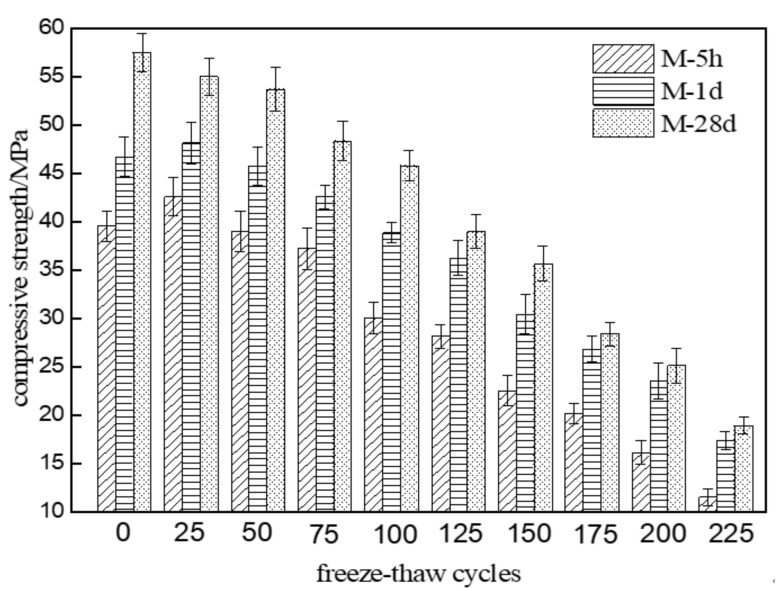
Compressive strength changes of MKPC mortar specimens with the number of freeze–thaw cycles in water.

Figure 3 shows the compressive strength changes of three different MKPC mortar specimens with an increasing number of freeze–thaw cycles in water. As can be seen from Figure 3, the compressive strength of M-5h and M-1d both rose to a peak value after 25 freeze–thaw cycles and then showed a fluctuating decreasing trend with further cycling. In contrast, the compressive strengths of M-28d showed a continuously fluctuating decreasing trend with an increasing number of freeze–thaw cycles. The compressive strength reached 75% of its initial values after 100 cycles for M-5 and 125 cycles for M-1d. On the other hand, the compressive strength of M-28d reached 75% of its initial values after 100 cycles. After 225 cycles, the compressive strengths were 29.1%, 37.2%, and 32.9% of their initial values, respectively. The results show that the compressive strength deterioration of MKPC mortar at 1-day hydration age is close to that of MKPC mortar at 28-day hydration age under freezing and thawing conditions in water.

**Figure 4:**
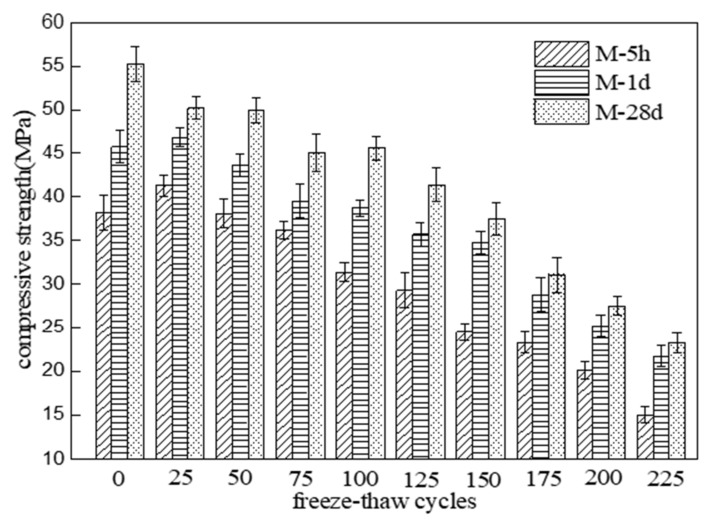
Compressive strength changes of MKPC mortar specimens with number of freeze–thaw cycles in 5% Na_2_SO_4_ solution.

Figure 4 shows the compressive strength changes of three different MKPC mortar specimens with the number of freeze–thaw cycles in a 5% Na_2_SO_4_ solution. The compressive strength of M-5h and M-1d gradually rose to a peak value after 25 freeze–thaw cycles and then showed a fluctuating decreasing trend with an increasing number of freeze–thaw cycles. In contrast, the compressive strength of M-28d showed a fluctuating decreasing trend with an increasing number of freeze–thaw cycles. The compressive strength reached 75% of its initial values after 125 cycles of M-5h and 150 cycles for M-1d. On the other hand, the compressive strengths of M-28d reached 75% of their initial values after 125 cycles. After 225 cycles, the compressive strengths of M-5h, M-1d, and M2-28d were 39.2%, 47.4%, and 42.1% of their initial values, respectively. The residual compressive strengths of M-1d and M-28d were similar. According to the results of Figure 3 and Figure 4, the compressive strength deterioration degree of MKPC mortar specimens under freezing and thawing conditions in sulfate solution is lower than that in water. The compressive strength deterioration degree of MKPC mortar at 1-day hydration age is close to that of MKPC mortar at 28-day hydration age under freezing and thawing conditions.

Under freeze–thaw conditions, MKPC mortar is not only subject to physical effects such as ice expansion pressure and water infiltration pressure but also to chemical processes such as continuous hydration of acid-base components [28,29] and hydrolytic loss of the main generated hydration product, MgKPO_4_·6H_2_O (MKP) [18]. Since MKPC has suitable adaptability to low temperatures [12], the continuous hydration of cement containing unreacted acid and base components in a supercooled water environment can still occur [30]. The hydration degree and structural strength of MKPC mortar specimens at a 5 h hydration age were low, with many capillary pores detected in their hardened bodies. When M-5h was placed in water under freeze–thaw conditions, a large amount of water infiltrated the capillary pores. At the initial stage of freeze–thaw cycling, sufficient water in the capillary pores enabled hydration of the MKPC paste to continue. The generated hydration product, MKP, then filled the internal pores of the mortar so that the structure of hardened mortar became more compact and its strength increased. With increasing freeze–thaw cycles, the capillary pores in the hardened mortar were continuously subjected to ice expansion pressure. The low initial structural strength caused the hardened MKPC mortar to bear fatigue stress and the capillary pores to become enlarged and connected. This effectively resulted in the structural deterioration and strength reduction in the hardened mortar. Due to its characteristics of rapid hardening and high early strength, the strength (especially the compressive strength) of the MKPC mortar specimens at a hydration age of 1 d was better than those at 5 h, and the structural compactness was also improved. At the initial stage of freeze–thaw cycling, the strength of the hardened body increased, also due to continued hydration. As the number of freeze–thaw cycles increased, the ice expansion pressure gradually degraded the structure and strength of the MKPC mortar. However, with its high initial structural strength, specimen M-1d showed greater resistance to fatigue stress due to water freezing expansion and significantly slower strength reduction than specimen M-5h. The MKPC mortar specimen at a hydration age of 28 d was fully hydrated. During freeze–thaw cycling, it was mainly subjected to pressure caused by water freezing in capillary pores. As capillary pores in the hardened body of M-28d were separated from each other by hydration products, the specimen was also subjected to osmotic pressure caused by supercooled water migration. With increasing freeze–thaw cycles, the combined effects of ice expansion pressure and osmotic pressure led to both structural deterioration and strength reduction in specimen M-28d. Due to M-28d’s high initial structural strength, its strength reduction rate was lower than that of M-5h. Due to the osmotic pressure caused by supercooled water migration, the strength reduction rate of M-28d was higher than that of M-1d.

The corrosion mechanism of cement mortar under rapid freeze–thaw cycling in salt solution was essentially the same as that in water. An advantage of the salt solution is its lower freezing point, which helps to reduce the ice expansion pressure in the hardened body. However, the hygroscopicity of salt can increase water saturation inside cement-based materials, which, when supercooled, will have more destructive effects upon freezing. In a low-temperature sulfate solution environment, sulfate radicals infiltrating into MKPC mortar specimens can react with Mg^2+^ to generate hydrated magnesium sulfate crystals, which fill the internal pores (see Section 3.7 SEM-EDS analysis for details). The structure of the MKPC mortar specimens was found to be more compact, making it difficult for the corrosive solution to continue to infiltrate into the specimen. Therefore, the strength loss of the MKPC mortar specimen subjected to freeze–thaw cycling was lower in sulfate solution than in water. Similarly, in the low-temperature sulfate solution environment, active calcium carbonate in the hardened MKPC specimens containing limestone sand could react with sulfate to form a new phase (see 3.6 XRD analysis for details) [31]. This new phase could then fill the pores of the hardened body, such that the strength loss in MKPC mortar specimens under freeze–thaw cycling was significantly lower in sulfate solution than in water.

### 3.4. Volume Deformation

**Figure 5:**
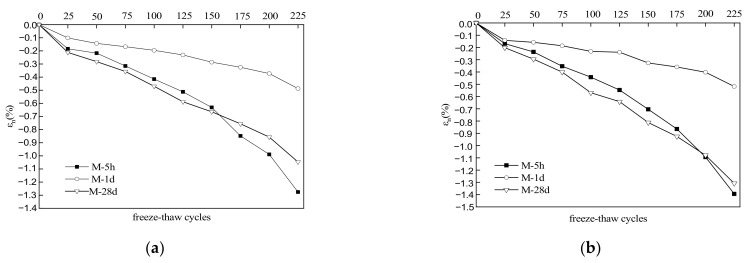
Trends of volume deformation of MKPC mortar specimens with number of freeze–thaw cycles. (**a**) Freezing and thawing in water. (**b**) Freezing and thawing in 5% Na_2_SO_4_ solution.

Figure 5 shows the trends in volume deformation of the MKPC mortar specimens with freeze–thaw cycling in water and 5% Na_2_SO_4_ solution. Figure 5a reveals that the volume of specimens gradually increased with cycling in water. Within the first 150 cycles, the volume expansion rates of the specimens decreased in the order of M-28d > M-5h > M-1d; afterward, they decreased in the order of M-5h > M-28d > M-1d. After 225 cycles, the volume expansion rates of specimens M-5h, M-1d, and M-28d were 1.276%, 0.487%, and 0.963%, respectively. Similarly, Figure 5b shows that the volume of the specimens increased with cycling in 5% Na_2_SO_4_ solution. Within the first 200 cycles, the volume expansion rates decreased in the order of M-28d > M-5h > M-1d; afterward, they decreased in the order of M-5h > M-28d > M-1d. After 225 freeze–thaw cycles, the volume expansion rates of specimens M-5h, M-1d, and M-28d were 1.394%, 0.518%, and 1.308%, respectively. A comparison of Figure 5a,b shows that the specimens under freeze–thaw had higher volume deformation in sulfate solution than in water. The results also show that hydration age had a significant impact on the deformation resistance. Furthermore, the specimen with a 1 d hydration age had better deformation resistance than those with a 28 d hydration age under freeze–thaw conditions.

Expansion deformation of the MKPC specimens after freeze–thaw cycles can be attributed to the high internal stress in the hardened body caused by the expansion of frozen solution in the capillary pores of the MKPC mortar. This deformation was higher in the salt solution than those in water due to the presence of sulfate ions (SO_4_^2−^) that can react with ions in the MKPC mortar to produce MgSO_4_·7H_2_O as well as active calcium carbonate (in hardened MKPC specimens containing limestone sand) to generate new phases. The MKPC mortar specimen at a hydration age of 5 h was found to have the highest expansion rate, which is due to its low initial structural strength. The MKPC mortar specimen at the hydration age of 1 d had relatively high initial structural strength, and so its volume expansion deformation was significantly lower. Although the MKPC mortar specimen at the hydration age of 28 d had a higher initial structural strength, its compact structure resulted in greater expansion stress upon ice expansion pressure and the generation of salt crystals. Its expansion deformation was even higher than that of MKPC mortar specimens at the hydration age of 1 d.

### 3.5. Water Absorption

**Figure 6:**
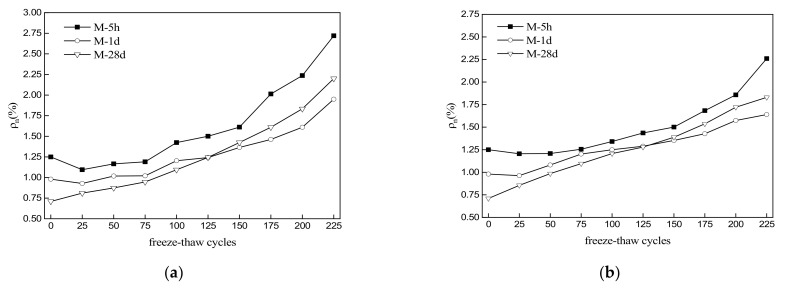
Trends of water absorption rates of MKPC mortar specimens with number of freeze–thaw cycles. (**a**) Freezing and thawing in water. (**b**) Freezing and thawing in 5% Na_2_SO_4_ solution.

Figure 6 shows the water absorption rates of the MKPC mortar specimens with freeze–thaw cycling in water and 5% Na_2_SO_4_ solution. The water absorption rates of specimens M-5h, M-1d, and M-28d before the freeze–thaw test were 1.25%, 0.98%, and 0.71%, respectively (Figure 6a). With freeze–thaw cycling in water, the water absorption rates of the specimens all showed fluctuating increasing trends. Within the first 125 freeze–thaw cycles, the water absorption rates decreased in the order of M-5h > M-28d > M-1d; afterward, they decreased in the order of M-5h > M-1d > M-28d. After 225 cycles, the water absorption rates of M-5h, M-1d, and M-28d were 2.72%, 1.95%, and 2.20%, respectively. In Figure 6b, the water absorption rates of the specimens show fluctuating increasing trends with freeze–thaw cycling in a sulfate solution. Within the first 125 freeze–thaw cycles in 5% Na_2_SO_4_ solution, the water absorption rates decreased in the order of M-5h > M-1d > M-28d; afterward, they decreased in the order of M-5h > M-28d > M-1d. After 225 freeze–thaw cycles, the water absorption rates of M-5h, M-1d, and M-28d were 2.26%, 1.64%, and 1.83%, respectively. A comparison of Figure 6a,b shows that the water absorption rates were significantly lower in sulfate solution than in water.

Water absorption is an indirect indicator of the structural compactness of cement-based materials. In MKPC mortar, higher water absorption rates are often related to greater open porosity and lesser compactness. With increasing hydration age, hydration products gradually fill the pores of the specimen, causing the open porosity and water absorption rate to gradually decrease. This was confirmed by the results of the present study; with freeze–thaw cycling, the water absorption rates of the MKPC mortar specimens gradually increased as ice expansion pressure and salt crystallization pressure deteriorated the pore structure of the hardened specimens. Although the initial open porosity of specimen M-1d was higher than that of M-28d, the open porosity of M-1d became lower than that of M-28d after > 125 freeze–thaw cycles. This indicates that deterioration of the pore structure of M-28d was more severe than that of M-1d during freeze–thaw cycling. The open porosity of the specimens under freeze–thaw conditions was lower in sulfate solution than in water, which suggests that salt crystallization and precipitation can improve the pore structure of hardened MKPC mortar to some extent.

### 3.6. XRD Analysis

**Figure 7:**
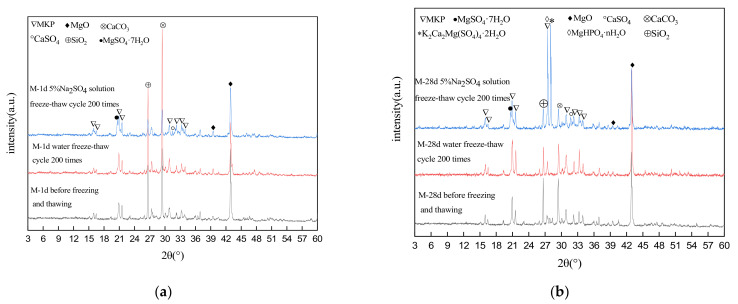
XRD patterns of MKPC mortar samples. (**a**) M-1d; (**b**) M-28d.

Figure 7a shows the XRD patterns of specimen M-1d under different freeze–thaw conditions. The positions of the main diffraction peaks in the XRD patterns of M-1d were largely the same under different freeze–thaw conditions. The characteristic peaks of unreacted MgO and the main hydration product MgKPO_4_·6H_2_O (MKP) were present in the spectra, as well as characteristic peaks of CaCO_3_ and SiO_2_ from limestone and river sand, respectively. In the XRD patterns of specimen M-1d subjected to 200 freeze–thaw cycles in sulfate solution, characteristic peaks of MgSO_4_·7H_2_O and CaSO_4_ were also found, indicating that sulfate from the solution reacted with some cations in the hardened MKPC to form a sulfate-containing hydrate crystal. Figure 7b shows the XRD patterns of M-28d under different freeze–thaw conditions, which have the characteristic peaks of unreacted MgO and the main hydration products, MgKPO_4_·6H_2_O (MKP), CaCO_3,_ and SiO_2_. In the XRD patterns of M-28d subjected to 200 freeze–thaw cycles in sulfate solution, characteristic peaks of MgSO_4_·7H_2_O, CaSO_4_, MgHPO_4_·nH_2_O, and K_2_Ca_2_Mg (SO_4_)_4_·2H_2_O were also found, indicating that sulfate from the solution reacted with cations in the MKPC-hardened body to form sulfate-containing hydrate crystals. In the low-temperature sulfate solution environment, the active calcium carbonate in the MKPC specimens containing limestone sand reacted with sulfate to form a new phase: K_2_Ca_2_Mg (SO_4_)_4_·2H_2_O. However, it has been confirmed in the literature that there are some amorphous hydration products [32,33], but we have not analyzed them by XRD. It is speculated that the content of the amorphous phase in the test sample is too small to be detected.

### 3.7. SEM-EDS Analysis

**Figure 8:**
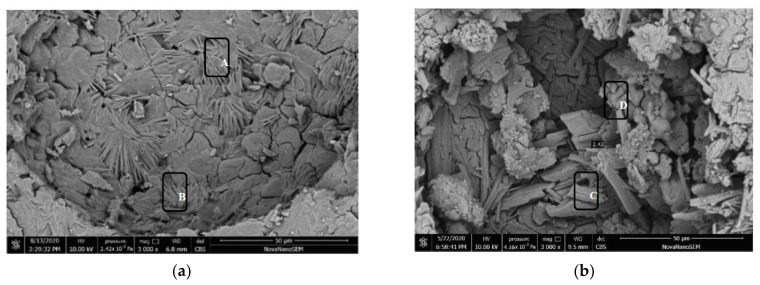

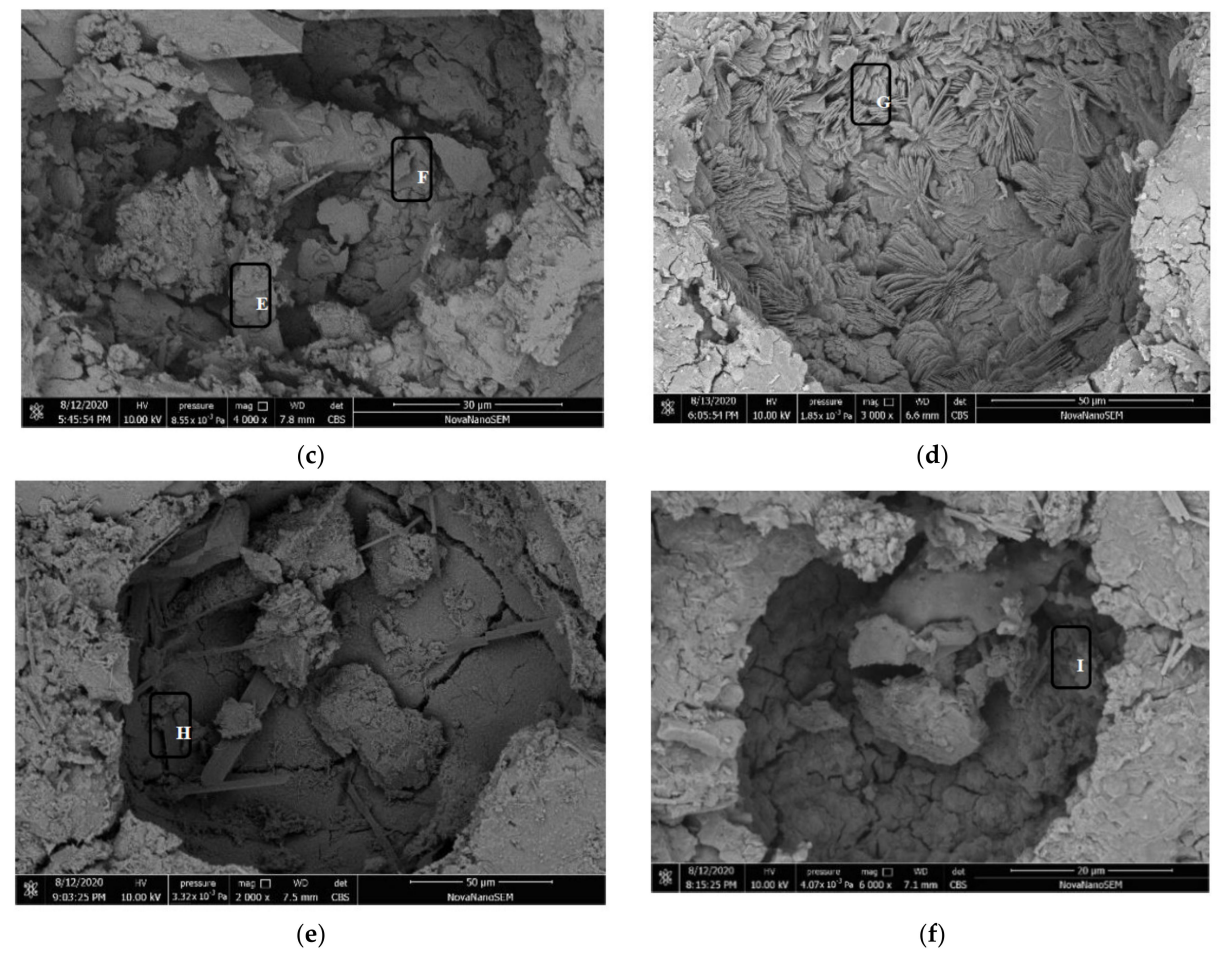
SEM patterns of MKPC mortar samples. (**a**) M-1d before freeze–thaw. (**b**) M-1d after 200 freeze–thaw cycles in water. (**c**) M-1d after 200 freeze–thaw cycles in sulfate solution. (**d**) M-28d before freeze–thaw. (**e**) M-28d after 200 freeze–thaw cycles in water. (**f**) M-28d after 200 freeze–thaw cycles in sulfate solution.

Figure 8a shows an SEM micrograph of specimen M-1d before freeze–thaw cycling. It shows that the pores are only partially filled with hydration products and that the structure is loose with many holes. Areas A and B are composed of elements O, Mg, P, and K. The molar ratio of P and K elements is close to 1:1, and the contents of O and Mg are high (Table 4). Referring to the XRD results, according to the chemical formula of MgKPO_4_·6H_2_O, it can be inferred that these two areas are composed of the main hydration product MKP and unreacted MgO from the MKPC paste. In addition, there is elemental Ca in area B, which came from limestone sand. Figure 8b shows the SEM image of specimen M-1d after 200 cycles in water. The hydration products are arranged in short columnar order, the crystal size has increased, and the degree of crystallization is significantly higher than that in Figure 8a. No obvious signs of corrosion are detectable on the crystal surface, but a small number of amorphous hydration products adhere to the crystal surface. An EDS analysis of crystal areas C and D is shown in Table 4. These areas were composed of Mg, P, and K elements at a molar ratio close to 1:1:1, indicative of the main hydration product of the MKPC paste, MKP. Figure 8c shows an SEM image of specimen M-1d after 200 freeze–thaw cycles in a sulfate solution. A high amount of hydration products fills the section pores. The EDS analysis results of areas E and F are shown in Table 4. The areas are composed of elemental O, Na, Mg, P, Cl, K, Ca, and S, where the molar ratio of Mg and P is close to 1:1. The Cl and Na were derived from the composite retarder, Ca came from the limestone sand, and S came from the solution. It is speculated that the two areas are the main hydration product MKP, which contains sulfate radicals. Figure 8d is an SEM image of the M-28d sample before freezing and thawing. It can be seen that the amount of hydration products filling the pores of the cross-section was significantly higher than that in Figure 8a, and the overall structure is dense. Combined with EDS analysis, area G was mainly composed of the hydration product MKP. Figure 8e shows an SEM image of specimen M-28d subjected to 200 freeze–thaw cycles in water. The hydration products in the pores are slender and needle-shaped with no obvious corrosion marks on their surfaces; however, there are obvious cracks on the pore walls, which can be attributed to ice expansion pressure. The EDS analysis results of area H are shown in Table 4, which indicate that the sample consisted of O, Na, Mg, P, K, and Ca. Figure 8f is an SEM image of specimen M-28d after 200 freeze–thaw cycles in a sulfate solution. The hydration products have short columnar shapes with no obvious corrosion traces on the crystal surfaces, but there are many attachments on the crystal surfaces. The EDS analysis results show that area I consisted of O, Na, Mg, P, Cl, K, Ca, and S elements (Table 4). The SEM results of the above four MKPC mortar samples after freeze–thaw cycling confirm that the surfaces of hydration products had not corroded and that cracks only existed in the pore walls. On the surfaces of the hydration products, amorphous substances were generated by continuous hydration, some of which contained sulfate radicals (specimens in sulfate solution).

### 3.8. TG-DTG

**Figure 9:**
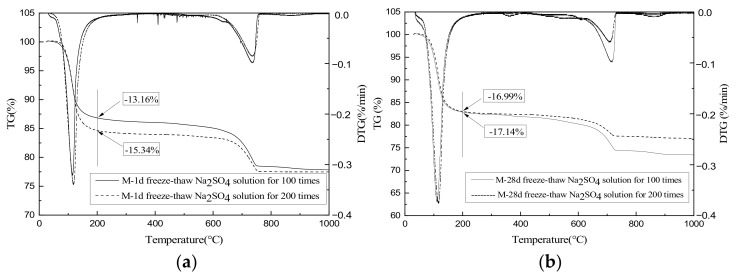
TG-DTG diagrams of MKPC mortar specimens. (**a**) M-1d. (**b**) M-28d.

Figure 9 shows the TG-DTG curves of the MKPC mortar specimens under different freeze–thaw conditions. The samples experienced an obvious mass loss at about 100 °C, which is due to the loss of crystal water from MgKPO_4_.6H_2_O [34]. This hydration product can completely lose its crystal water before 200 °C is reached [35]. The mass-loss rates of M-1d subjected to 100 and 200 freeze–thaw cycles in sulfate solution at 200 °C were 13.16% and 15.34%, respectively (Figure 9a). Because the specimens were subjected to freeze–thaw cycling after only one day of hydration, they were not fully hydrated and, thus, contained many unreacted phosphates and MgO. Research [22] has proven that the reaction between phosphate and MgO in MKPC can occur even at low temperatures, which is confirmed by the above test results—the amount of MKP increased with time in the low-temperature environment. At 200 °C, the mass-loss rates of specimen M-28d subjected to 100 and 200 freeze–thaw cycles in sulfate solution were 17.14% and 16.99%, respectively; that is, 30.24% and 10.76% higher than those of M-1d under the same conditions. These results further confirm that the hydration of MKPC mortar of 1 d of hydration was incomplete. Since specimen M-28d was sufficiently hydrated, its mass-loss rates after 100 and 200 freeze–thaw cycles in sulfate solution were largely the same.

## 4. Conclusions

This paper studied the freeze–thaw resistance of MKPC mortar of various hydration ages in water and sulfate solution. The results show that:

(1) After 225 freeze–thaw cycles in water, the flexural strengths of specimens M-1d and M-28d were 36.7% and 29.0% of their initial values, respectively, while the compressive strengths were 37.2% and 32.9% of their initial values. After 225 cycles in sulfate solution, the flexural strengths of M-1d and M-28d were 45.1% and 36.0% of their initial values, respectively, and the compressive strengths were 47.4% and 42.1% of their initial values. The results show that, under freeze–thaw conditions, the degree of strength reduction in MKPC mortar with 1 d hydration is similar to that with 28 d hydration. Furthermore, the strength reduction is lower in sulfate solution than in water.
(2) After 225 freeze–thaw cycles, the volume expansion rates of specimens M-1d and M-28d were 0.487% and 1.047% in water and 0.518% and 1.308% in sulfate solution, respectively. Hence, MKPC mortar under freeze–thaw conditions has better deformation resistance with 1 d hydration than with 28 d hydration.
(3) Although the open porosity of specimen M-1d was higher than that of M-28d before freeze–thaw testing, it became lower after 125 freeze–thaw cycles. This indicates that freeze–thaw cycling more strongly degraded the pore structure of M-28d than that of M-1d. The open porosity of MKPC mortar specimens subjected to freeze–thaw cycling was lower in sulfate solution than in water, which proves that salt crystallization and precipitation can improve the pore structure of hardened MKPC bodies to some extent.

In conclusion, the anti-water freezing and sulfate freeze–thaw resistance of MKPC mortar specimens with a hydration age of 1 d is better than those of MKPC mortar specimens with a hydration age of 28 d, which reflects its rapid acquisition of environmental erosion resistance and is suitable as a rapid repair material.

## Figures and Tables

**Table 1:** Oxide composition of the dead burned magnesia powders.

Oxide Species	MgO	SiO_2_	CaO	Fe_2_O_3_	Al_2_O_3_	Na_2_O	TiO_2_	Other
Content (%)	91.85	3.68	3.14	0.865	0.17	-	-	0.285

**Table 2:** The physical indexes of sand.

Type	Speciation	Fineness Modulus	Clay Content (%)	Bulk Density (kg/m^3^)	Particle Gradation
Ordinary river sand	Medium sand	2.53	0.8	1450.00	Zone II (JGJ52-2006)
Limestone sand	Coarse sand	3.65	0.00	1460.00	Zone II (JGJ52-2006)

**Table 3:** Mix ratio of magnesium potassium phosphate cement (MKPC) mortar.

Code	Mass Ratio/%					
	W_MgO_/ W_KH2PO4_	W_CR_/ W_MKPC_	W_S_/W_MKPC_	W_RS_/W_S_	W_LS_/ W_S_	W_w_/ W_MKPC_
M	150	8	150	33.3	66.7	17

Note: MKPC refers to magnesium potassium phosphate cement, RS refers to river sand, LS refers to limestone sand, S refers to river sand and limestone sand, W refers to water.

**Table 4:** Distributions of elements in different regions of the MKPC mortar samples.

Element		O	Mg	S	P	Cl	K	Ca	Na
Atomic percentage (%)	Area A	68.45	12.52	-	8.79	-	10.24	-	-
	Area B	68.72	10.70	-	9.47	-	9.78	1.33	-
	Area C	70.11	10.96	-	9.87	-	9.06	-	-
	Area D	72.92	8.95	-	9.59	-	8.54	-	-
	Area E	73.45	8.56	2.45	7.53	0.13	5.93	1.52	0.43
	Area F	74.33	6.41	1.99	6.12	-	6.57	3.70	0.88
	Area G	72.3	10.36	-	8.12	-	9.22	-	-
	Area H	73.52	8.43	-	6.95	-	6.07	2.11	2.92
	Area I	66.35	7.47	2.56	8.85	0.35	9.55	2.86	2.01

## Data Availability

Data are contained within the article.
